# Factors Associated with RANTES, EMMPIRIN, MMP2 and MMP9, and the Association of These Biomarkers with Cardiovascular Disease in a Multi-Ethnic Population

**DOI:** 10.3390/jcm11247281

**Published:** 2022-12-08

**Authors:** Laureen Yi-Ting Wang, Chuen Seng Tan, Mitchell K. P. Lai, Saima Hilal

**Affiliations:** 1 National University Heart Centre, National University Hospital Singapore, Singapore 119074, Singapore; 2 Saw Swee Hock School of Public Health, National University of Singapore and National University Health System, Singapore 117549, Singapore; 3 Department of Pharmacology, Yong Loo Lin School of Medicine, National University of Singapore, Singapore 117600, Singapore; 4 Memory, Aging and Cognition Centre, National University Health System, Singapore 117600, Singapore

**Keywords:** biomarkers, coronary artery disease, stroke

## Abstract

**Highlights:**

**What are the main findings?**

- This is the first multi-ethnic population-based study to use these serum biomarkers for preventive strategy.
- No association was found between RANTES, EMMPRIN, MMP2, and MMP9 with CVD.

**What is the implication of the main findings?**

- Our research improves the understanding of inflammatory biomarkers in the cardiovascular field. Currently, these biomarkers are ineffective for risk stratification or diagnosis when used as a single indicator.
- Prevention of CVD still requires a comprehensive evaluation of CVD risk factors.

**Abstract:**

Background: The growing cardiovascular disease (CVD) epidemic calls for further research to identify novel biomarkers for earlier detection and as potential therapeutic targets. Biomarkers Regulated on Activation, Normal T Cell Expressed and Secreted (RANTES), extracellular matrix metalloproteinase inducer (EMMPRIN), and matrix metalloproteinases (MMP-2, and MMP-9) are linked to proatherogenic and proinflammatory pathways of CVD development, the majority of which are coronary artery disease (CAD) and stroke. We evaluated potential factors affecting these four biomarkers and established their association with CVD. Methods: This is a cross-sectional analysis using a nested case-control design involving 580 participants aged 21–75 years from the prospective multi-ethnic cohort study. A total of 290 CVD cases and 290 age-and sex-matched controls were identified. All participants underwent interviews, health screenings, and provided blood samples, including biomarkers RANTES, EMMPRIN, and MMPs. CVD was defined based on previous medical history. Results: The average age of the participants was 55.7(SD = 10.3) years of age, and 34.6% were female. Arrhythmia history and low-density lipoprotein (LDL) levels were significant factors of logEMMPRIN (β = −0.124 [−0.245, −0.003] and β = 0.111 [0.0, 0.191], respectively). Only female sex (β = 0.189 [0.078, 0.300]) for logRANTES and age (β = 0.033 [0.010, 0.055]) for logMMP-2 and logMMP-9 were significant. The Indian ethnicity (β = 0.192 [0.048, 0.335]) and highly sensitive C-reactive protein (hs-CRP) levels (β = 0.063 [0.011, 0.116]) were statistically significant for logMMP-9. No association was detected between biomarkers and CVD. Conclusions: In this multi-ethnic study cohort, RANTES was associated with sex, EMMPRIN was associated with a history of arrhythmia and LDL levels, MMP-2 with age, and MMP-9 with ethnicity and hs-CRP levels. The biomarker serum levels were not associated with CVD.

## 1. Introduction

Cardiovascular disease (CVD) is one of the major causes of death, accounting for over 19 million deaths annually and approximately 31% of worldwide deaths [1]. CVD encompasses a group of diseases affecting the heart and vasculature of which coronary artery disease and stroke are the most common forms [2]. Heart disease and stroke share similar risk factors, such as diabetes mellitus, hypertension, hyperlipidemia, unhealthy diet, and tobacco use, resulting in the underlying pathophysiology of atherosclerosis [3]. It is well established that chronic inflammation is the key driver of atherosclerosis and cardiovascular events [4]. Atherosclerosis can be a silent process with stable plaques filled with chronic inflammatory infiltrates or unstable with “active” inflammation and ischemic symptoms, resulting in AMI, transient ischemic attacks, or stroke. Traditional biomarkers, such as low-density lipoprotein and glycated hemoglobin (HbA1c), while essential, are only part of the atherosclerotic pathway, do not address the residual risks, and exclude a significant population of those who present with CVD events without traditional risk factors [5]. Relying only on conventional risk factors impedes the ability to identify high-risk individuals long before the development of CVD events. As a result, in recent years, novel peripheral inflammatory biomarkers have been proposed for earlier risk stratification and as potential specific targets in inflammatory pathways before the development of CVD [6].

Regulated on Activation, Normal T cell Expressed and Secreted (RANTES), also known as chemokine ligand (CCL5), is a proinflammatory cytokine generated by activated T cells, macrophages, and platelets that plays a role in atherosclerosis pathogenesis [7,8]. Higher RANTES expression is seen in vasculature that is vulnerable to high shear stress of blood flow [9]. However, the association of RANTES with CVD is contradictory and limited data suggests that low-baseline plasma RANTES levels were an independent predictor of cardiac death in males referred for coronary angiography [9], whereas elevated plasma RANTES levels were reported in acute coronary syndrome [7].

Extracellular matrix metalloproteinase inducer (EMMPRIN) is a cell surface glycoprotein of the immunoglobulin superfamily. EMMPRIN induces MMP-2 in smooth muscle cells and MMP-9 in monocytes. In-vitro studies show that EMMPRIN surface expression is enhanced in hypoxia/ischemia and normalizes after successful therapy [10]. EMMPRIN has been shown to promote plaque instability by inducing extracellular matrix degradation and MMP synthesis, though the mechanism remains unclear [11]. Small sample studies (*n* < 100) show that EMMPRIN levels were elevated in myocardial infarction [12,13] and its up-regulation in neural injury (degeneration and gliomas) in mouse models [10]. However, it remains unclear whether elevated levels of EMMPIRIN are detrimental or advantageous for CVD.

Extracellular Matrix Metalloproteinases (MMPs) are a family of endopeptidases produced by macrophages and neutrophils that regulate a variety of physiological and pathological processes, such as tissue remodeling, inflammation, aging, and cancer. Consequently, dysregulated activity can result in pathology [14]. In the context of CVD, MMP-2, and MMP-9 are implicated in destabilizing atherosclerotic plaques and increase the risk of ischemic strokes and CAD [15,16,17]. MMP-9 has been associated with both cardiovascular and cerebrovascular death, as well as heart failure [18]. MMP-2 is also elevated post myocardial infarction [19] and is an independent predictor of all-cause mortality in post-acute coronary syndrome [20]. However, the source of plasma MMP-9 cannot be ascertained as the elevated levels may originate from other vascular beds or diseases. 

Despite a growing body of research demonstrating the importance of the inflammatory process in the progression of CVD, it remains unclear which factors activate these inflammatory indicators. Furthermore, the majority of the research is conducted in an acute disease state. There have been relatively few population studies exploring the utility of these four biomarkers to improve the existing risk-stratification metrics for CVD and the selection of individuals for preventive therapies. In this cross-sectional analysis of a nested case-control study conducted within a large multi-ethnic Singaporean population, we aim to evaluate factors associated with these four biomarkers, RANTES, EMMPRIN, MMP-2, and MMP-9, and establish their association with CVD.

## 2. Methods

### 2.1. Study Sample

This nested case-control study was derived from the prospective multi-ethnic cohort (MEC) study. The MEC cohort was developed by merging two cohorts—Singapore Prospective Study Program (SP2) and the Singapore Cardiovascular Cohort Study (SCCS2) from 2004 to 2007–with further participant recruitment via public outreach methods from 2007 to 2010 [21]. A total of 14,465 Singaporeans and long-term residents aged 21 to 75 years of Chinese, Malay, and Indian ethnicity formed the cohort. At baseline, the participants completed interviewer-administered structured questionnaires that included sociodemographic, lifestyle practices, family, and medical history, as well as health examinations and blood taking. From 2011 to 2016, a total of 6101 participants were revisited, and participants’ blood samples and medical history were collected.

**Figure 1:**
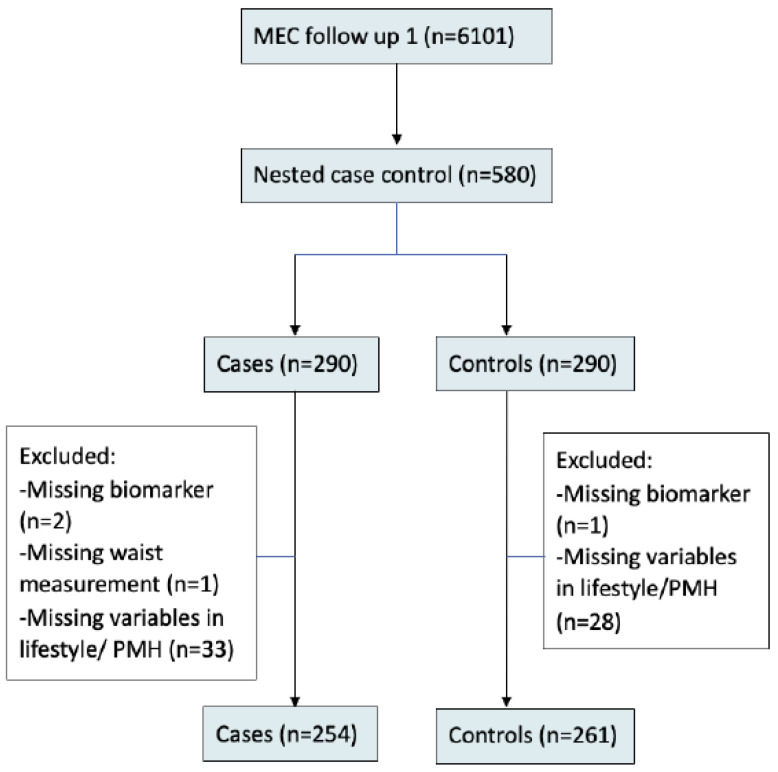
Flow chart of the study population.

Of the 6101 participants, 580 (290 cases and 290 age and sex matched controls) MEC participants from the follow-up visit were enrolled in this study. Cases of CVD were defined based on self-reported IHD (N = 150), which is defined as a previous physician or angiogram diagnosis, previous angioplasty or bypass surgery, and abnormal electrocardiogram (ECG) findings of q waves suggestive of IHD (N = 47). Cases also included those with self-reported TIA or stroke (N = 57). The case status was further validated by use of aspirin medication. Controls were individuals without any history of CVD. Subsequently, 515 participants (254 cases and 261 controls) were included in the final analysis, as those with incomplete biomarker investigations were excluded from the analysis (Figure 1). 

Ethics approval was obtained from the National University of Singapore Institutional Review Board. Signed informed consents were obtained from all participants by bilingual study coordinators, in the participants’ preferred language prior to recruitment.

### 2.2. Assessment of Biomarkers

At the follow-up visit of the cohort study (which is the recruitment visit of the case-control study), participants’ non-fasting blood was collected into serum separating tubes and centrifuged at 2000× *g* for 10 min at 4 °C, followed by extraction of the top serum layer and storage at −80 °C until use. Serum RANTES, EMMPRIN, MMP-2, and MMP-9 (Quantikine®, Catalogue numbers DEMP00, MMP200, and DMP900, R&D Systems, Inc. Minneapolis, MN, USA) concentrations were measured using quantitative sandwich enzyme-linked immunosorbent assays (ELISAs) following manufacturer’s instructions. Before adding serum samples to the antibody-coated plate, serum samples were diluted 3-, 10-, 20-, and 100-fold in calibrator diluent solution for RANTES, EMMPRIN, MMP-2, and MMP-9, respectively. Detection was conducted by adding the stabilized chromogen (tetramethylbenzidine), and color development was stopped after 10 to 30 min by adding an acidic stop solution. Absorbance was measured at 450 nm on a microplate reader (BioTek, Winooski, VT, USA) with background subtraction at 570 nm, and a standard curve range was generated for each biomarker assay. Sample concentrations read from the corresponding standard curves were multiplied by the respective dilution factors to obtain the actual biomarker concentrations in serum. All blood samples were measured in duplicate and analyzed blinded to subject characteristics and clinical status. Besides the biomarkers, the blood samples taken were also assessed for creatinine, estimated glomerular filtration rate (eGFR), HbA1c, high-sensitive C-reactive protein (hs-CRP), and cholesterol levels using a clinical chemistry analyzer (DxC 600, Beckman Coulter, Brea, CA, USA).

### 2.3. Assessment of Covariates

Conventional cardiovascular risk factors, such as diabetes mellitus, hypertension, hyperlipidemia, and family history, of CVD were obtained from the questionnaires. The questionnaires also had detailed past medical history, such as chronic kidney disease (CKD), gout, arrhythmia, and sedentary time per week. The participant’s medical history was recorded as “Yes” if the condition had been diagnosed and “No” if the condition had never been diagnosed. Medication usage (lipid lowering medications and antihypertensives) were self-reported and verified if prescriptions were brought by the participants. Sedentary time was calculated as the sum of hours spent resting or sitting during weekdays and weekends per week. Smoking history was classified as “Yes” for current or previous smokers and “No” for those who had never smoked. Sedentary time was calculated as the sum of hours spent resting or sitting by the participants during the weekdays and weekends. Based on reported answers, the Framingham risk score (FRS) for CVD was calculated. Higher risk scores indicate a higher risk for major adverse cardiovascular events, such as CAD, stroke, and cardiac-related mortality [22]. The presence of metabolic syndrome was assessed using the adult treatment panel III report (ATP III) criteria from the National Cholesterol Education Program [23], which incorporates waist circumference, triglycerides, high-density lipoprotein, blood pressure, and hyperglycemia. Height was taken using a stadiometer (SECA 200 series) and weight was measured on a digital scale after participants emptied their pockets (SECA 700 series). Body mass index (BMI) was obtained by dividing the participants’ weight in kilograms by their height in meters squares. The waist circumference was measured between the last rib and the iliac crest. After participants had rested for 5 minutes, an average of two automated blood pressure readings (Dinamap Carescape V100) were collected in a calm conducive setting. A third reading was obtained if the systolic blood pressure (SBP) differed by more than 10 mm Hg or the diastolic blood pressure differed by more than 5 mm Hg. A 10 lead electrocardiogram (ECG) was performed in the supine resting position (Nihon Kohden ECG-1350 K).

### 2.4. Statistical Analysis

Demographic parameters, cardiovascular risk factors, and serum levels of the biomarkers RANTES, EMMPIRIN, MMP2, and MMP9 were summarized using mean ± standard deviation (SD) for continuous data and frequency and percentages (%) for categorical data.

Normality was assessed through histogram plots visually followed by the Shapiro-Wilk test.

Comparisons between cases and controls were evaluated using chi-square for categorical variables and Student’s *t*-tests for continuous variables. As RANTES, MMP-2, MMP-9 and EMPIRIN were not normally distributed, the logarithmically transformed values that improved normality were used in the models. The first objective was to find potential factors associated with each of these four biomarkers using multiple linear regression. We build up models using the forward stepwise approach. For Model 1.1, forward stepwise selection was performed where the p-value thresholds for variables to enter and exit the model were 0.1 and 0.2. Sensitivity analysis was conducted using subsequent adjustments from Models 1.2 to 1.4 to take into account other potential competing variables of the biomarkers. In Model 1.2, age, gender, and ethnicity were added to the model if these variables did not already enter the model. In Model 1.3, the models differed slightly for each biomarker because additional variables were added based on previously published literature as potential factors of the individual biomarker [9,24,25,26,27,28,29,30,31]. For RANTES, Model 1.3 was further adjusted for history of gout, metabolic syndrome, body mass index (BMI), serum creatinine, and hs-CRP. For EMMPRIN, Model 1.3 was further adjusted for CAD, arrhythmia history, stroke, CKD, family history, sedentary time, low-density lipoprotein (LDL), and hs-CRP. For MMP-2, Model 1.3 was adjusted for CAD, sedentary time, and LDL, and for MMP-9, Model 1.3 included CAD, BMI, and LDL. The final model1.4 for all four biomarkers was the same and incorporated all the variables listed in Model 1.3. In addition, sensitivity analysis was conducted for MMP-2 and MMP-9 to account for the menopausal status in women due to the effect of the menstrual cycle on MMPs [32].

Logistic regression was used to determine the association of these biomarkers with CVD cases. The first model, Model 2.1 was adjusted for significant biomarker variables identified from the final Model 1.4 of objective 1 with *p* < 0.05 as they may be potential confounders. Model 2.2 was adjusted for age, gender, and ethnicity if they were not in the first model. Baseline variables identified as significantly different between cases and controls from the earlier chi-square and Student’s t-tests were added to Model 2.3 if they were not present in the prior models. The final Model 2.4 incorporated all factors that appeared in Model 2.3 of the biomarkers but were shared by all four biomarkers. All statistical analyses were performed using STATA 17, with a 2-sided *p*-value of 0.05 indicating statistical significance.

## 3. Results

### 3.1. Participant Characteristics

Of the 515 participants analyzed (Table 1), 178 were women (34.6%), and the average age was 55.7 years (SD 10.31). The majority were of Chinese ethnicity (64.3%), followed by Indians (21.6%) and Malays (3.5%). A total of 27.2% were current or former smokers, 37.1% had hypertension, 38.3% had hyperlipidemia, and 16.3% had type 2 diabetes mellitus. Almost a third of the population had metabolic syndrome. Gout was prevalent in 7.4% and CKD in 1.2%. Approximately a quarter had a family history of CVD.

### 3.2. Factors Associated with the Biomarkers

**Figure 2:**
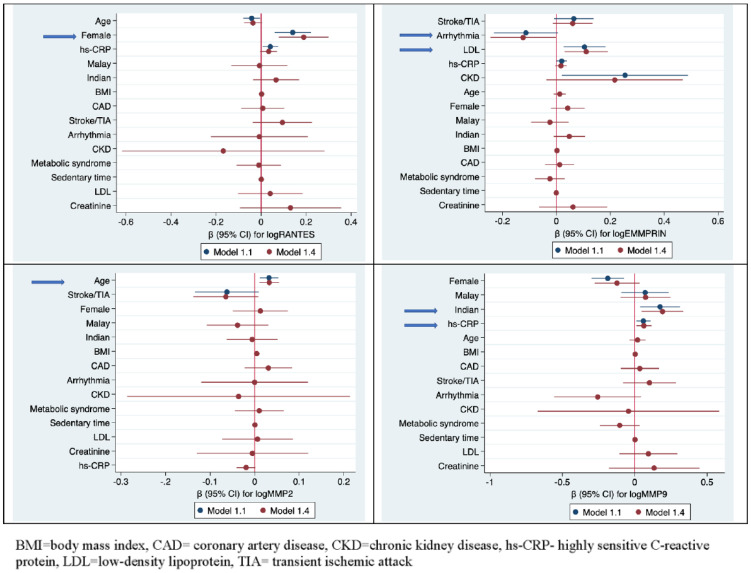
Forest plot for factors associated with the biomarkers.

Table 2 shows the factors associated with the individual biomarkers, and Figure 2 illustrates the initial and final models for each biomarker on the forest plot.

#### 3.2.1. RANTES

RANTES was significantly associated with younger age, female sex, Indian ethnicity, higher BMI, lower FRS, and increasing hs-CRP according to univariate analysis (Model 1.0). After stepwise forward selection, the variables age, sex, and hs-CRP entered the model and were significantly associated with RANTES (Model 1.1). In the second model (Model 1.2), which additionally included ethnicity, the variables that entered Model 1.1 remained significant. However, after adding in variables with previously reported associations with RANTES (i.e., CKD, serum creatinine, BMI, and metabolic syndrome), only younger age (β = −0.043; 95% CI: −0.08, −0.007) and female sex (β = 0.212; 95% CI: 0.109, 0.314) remained significant (Model 1.3). In the final model (Model 1.4), only female sex remained significant (β = 0.189; 95% CI: 0.078, 0.300).

#### 3.2.2. EMMPRIN

Univariate analysis revealed that a history of stroke, Indian ethnicity, increasing LDL levels, and higher hs-CRP were significantly associated with EMMPRIN. Using stepwise forward selection, the first model contained the history of arrhythmia, stroke, CKD, LDL, and hs-CRP (Model 1.1) of which the last three are significant. In the second model (Model 1.2), only a history of CKD and LDL remained significant after adjusting for age, sex, and ethnicity. Subsequent adjustment for sedentary behavior (Model 1.3) did not attenuate this association. A history of arrhythmia (β = −0.124; 95% CI: −0.042, −0.066) and LDL levels (β = 0.111; 95% CI: 0.030, 0.191) were significantly correlated with EMMPRIN levels in the final model (Model 1.4).

#### 3.2.3. MMP-2

Univariate analysis showed that increasing age and Framingham risk scores were significantly associated with MMP-2 levels. In the first model (Model 1.1), histories of stroke and age entered the model by forward stepwise selection, with age being the only significant factor. In the second model (Model 1.2), which additionally included sex and ethnicity, age remained significantly correlated with MMP-2. Despite adjustments in Models 1.3 and 1.4, no new associations were found, nor was the effect of age attenuated (β = 0.033; 95% CI: 0.010, 0.055). Taking into account the menopausal status of women, sensitivity analysis did not yield different conclusions (Supplementary Materials).

#### 3.2.4. MMP-9

Univariate analysis showed that female sex, Indian ethnicity, serum creatinine, and hs-CRP were significantly associated with MMP-9 levels. The first model (Model 1.1) had sex, ethnicity, and hs-CRP entering the model with forward stepwise selection with sex and ethnicity significantly associated with MMP-9. In the second model (Model 1.2), after adjustment for age, sex and ethnicity, hs-CRP was significantly correlated with MMP-9 levels. Subsequent adjustment for other previously reported associations did not attenuate the significance of female sex and hs-CRP levels in Model 1.3. In the final model (Model 1.4), Indian ethnicity (β = 0.192 [CI 0.048, 0.335]) and hs-CRP (β = 0.063 [CI 0.011, 0.116]) were significant. Sensitivity analysis for menopausal status did not change the overall findings (Supplementary Materials).

### 3.3. Association of Biomarkers with CVD

On univariate analysis, the mean values of all four biomarkers did not differ statistically between those with and without CVD. Variables that significantly associated with each biomarker from Model 1.4 were added in the first logistic regression model for CVD, Model 2.1, and subsequent adjustment for age, sex, and ethnicity in Model 2.2 did not alter the findings for all 4 biomarkers. Model 2.3 included significant factors for CVD identified in Table 1.

Except for smoking, those with CVD showed a significantly higher proportion of conventional risk factors of diabetes, hyperlipidemia, and hypertension than those without. Those with CVD were also more likely to have higher Framingham scores, increased abdominal circumference, BMI, and systolic blood pressure (SBP). Serum creatinine, hs-CRP, and HbA1c were significantly elevated in participants with CVD compared to those without CVD. Other risk factors, such as metabolic syndrome, gout, chronic kidney disease, and family history of CVD did not differ significantly between the two groups. Adjustments for these potential confounders from Table 1 did not alter our findings. In the final model, Model 2.4, no significant association was found between RANTES (OR = 0.934), EMMPRIN (OR = 1.348), MMP-2 (OR = 0.836) and MMP-9 (OR = 1.044) with CVD (Table 3).

## 4. Discussion

In this multi-ethnic study cohort, we found that RANTES is associated with sex, EMMPRIN is associated with a history of arrhythmia and LDL levels, MMP-2 with age, and MMP-9 with ethnicity and hs-CRP levels. No associations were observed between serum levels of RANTES, EMMPIRIN, MMP-2, and MMP-9 with CVD.

In one of the few population studies on RANTES, Tetsuya et al. found that RANTES levels were associated with metabolic syndrome in their cohort of 210 middle-aged (40.9 years SD 9.5) healthy Japanese males [33]. Age, interleukin 6 (IL-6), and plasma platelet-derived microparticles (PDMP) were found to be predictive of RANTES on multivariate analysis. However, our study did not detect a correlation between metabolic syndrome and RANTES, and only the female sex positively correlated with RANTES levels. Possible reasons for this include that our study population is more diverse, with a greater age range, inclusive of more ethnicities and both sexes. In the MONICA/KORA Ausburg case-cohort population investigation, no correlation between RANTES and incident cardiac coronary events was detected. The study also assessed the influence of RANTES polymorphisms and found no correlation with coronary events. They also observed no relationship between RANTES levels in carotid plaques and future coronary heart disease risk [34]. This is consistent with the findings in this study that found no correlation between RANTES levels and either combined CVD or CAD alone, which indicate that plasma RANTES levels may not be a suitable biomarker for assessing CVD risk in humans

Our study reported that the history of arrhythmias was associated with decreasing EMMPRIN levels, whereas increasing LDL levels remained positively correlated with EMMPRIN levels. Studies from mouse models have shown that the mice fed with a high-fat diet exhibited increased EMMPRIN expression, and in mice given neutralizing antibodies to EMMPRIN, lipid-filled atherosclerotic lesions were reduced in the aorta [35]. Limited data regarding EMMPRIN and history of arrhythmias or palpitations reported that in a small sample of patients with cryptogenic stroke or TIA who subsequently had subclinical atrial fibrillation, growth differentiation factor (GDF-15) correlated with premature atrial contractions and subclinical atrial fibrillation but not EMMPRIN [36]. This is in line with our findings where EMMPRIN was not associated with CVD. There is a need for additional research on arrhythmias and EMMPRIN to analyze this relationship.

MMP-2 and MMP-9 have been extensively investigated in the context of vascular remodeling and angiogenesis. In a community study with 447 non-hypertensive healthy individuals without any history of symptomatic CVD, aortic pulse wave velocity, mean arterial pressure, and CRP correlated positively with MMP-9 levels [37]. MMP-9 levels are thought to enhance inflammation and vascular wall degradation, resulting in greater arterial stiffness and a potentially increased risk of hypertension and other CVD. Our study corroborates these findings by reporting that hs-CRP was associated with log MMP-9 levels. The other significant finding in our final adjusted model for log MMP-9 was that Indian ethnicity was associated with higher MMP-9 levels than Chinese (*p* = 0.009). Besides MMP-9 levels, Indian ethnicity was also associated with RANTES and EMMPRIN in univariate analysis. This may indicate a higher inflammatory state in Indians. Among the ethnicities in Singapore, the Indian population has the highest rates of insulin resistance and IHD, which may explain the pro-inflammatory state [38,39]. This finding may also be attributable to ethnic-related genetic polymorphisms of the MMP-9 promoter, as well as sociocultural differences, and ethnic inequalities. However, these variables were not examined in this study. Similarly, in an Iranian study, serum MMP-2 and MMP-9 were elevated in patients with recent symptoms suggestive of CAD and proven elevated coronary artery calcium (CAC) scores compared to healthy controls [29]. On the other hand, sub-analysis of our CAD cases (self-reported IHD and previous angiography or angioplasty) revealed no association with MMP-2 or MMP-9 levels. This may be due to the variation in how CAD cases are defined, as well as the fact that our participants were also not acutely symptomatic. Moreover, CAC score is not only prognostic of coronary atheroma burden but also other systemic atherosclerotic sites in the vascular tree and myocardium; hence, sources of these MMPs cannot be established [40].

Although we found no association between RANTES, EMMPRIN, MMP-2, MMP-9 and CVD, our study adds to the current understanding of vascular biomarkers. Our findings are not surprising nor do they contradict previous research, as the majority of the positive findings were based on animal studies and organ or tissue-specific expression of these biomarkers, which does not necessarily translate to a rise in systemic levels. Moreover, the results of inflammatory inhibition therapy and their effect on vascular biomarkers and CVD outcomes in clinical trials have been varied. For example, low-dose methotrexate used in cardiovascular inflammatory reduction trial (CIRT) did not lower IL-1β, IL-6, CRP levels, or reduce cardiovascular events in patients with stable atherosclerosis [41]. On the other hand, canakinumab anti-inflammatory thrombosis outcomes study (CANTOS) showed that Canakinumab, a selective inhibitor of IL-1β, reduced hs-CRP, IL-6 and cardiac events in patients with a previous history of AMI [42] In particular, individuals with persistently increased hs-CRP levels despite treatment with Canakinumab, indicating a high residual risk, were more likely to experience cardiac events. Other research on potential clinical applications of inflammatory biomarkers include neutrophil-to-lymphocyte ratio (NLR), platelet-to-lymphocyte ratio (PLR) and systemic inflammation index (SII), where elevated levels are associated with poorer post operative outcomes in acute limb ischemia, arterio-venous fistula patency, post operative delirium in vascular surgery patients, and post discharge outcomes in AMI [43,44,45,46]. More work is, therefore, needed to understand the role of these inflammatory biomarkers for cardiac events and diverse systemic vascular effects.

These biomarkers are frequently measured in studies at an acute CVD event or one-time point, but these plasma biomarker levels are dynamic, with variations over time and context that must be considered, especially for CVD, which includes a wide range of disease states, such as subclinical atherosclerosis development versus AMI, and TIA versus stroke [47]. In a heterogeneous population setting, multiple systemic processes may occur simultaneously; some may result in an increase in biomarker expression in one organ and yet a decrease in expression in another. MMP-9, for instance, is both a proximal biomarker, defined as one that has a direct impact on target disease pathology, such as cardiac remodeling post-infarction, as well as a distal biomarker, defined as a more systemic and less organ-specific modifying process, such as atherosclerosis, or periodontitis and chronic inflammation from rheumatoid arthritis [48]. Population-based research related to these biomarkers has shown inconsistent results. Lower circulating levels with poor stability and less refined assays compared to hs-CRP may make these biomarkers unsuitable for regular use in predicting CAD in non-acute settings or less symptomatic individuals [49]. There is still no direct evidence linking these inflammatory biomarkers with future CVD in asymptomatic individuals. While these biomarkers are involved in specific pathologies and are beneficial when researching tissue-specific molecular expression or acute symptomatic disease states, they are unlikely to be useful biomarkers for risk stratification or prognosis when used as a single indicator.

This study has several potential limitations. As participants were drawn from the MEC population cohort, the population is more heterogeneous than many of the experimental biomarker investigations that have been previously described. Therefore, any substantial effect in this general population setting may be small. A larger sample size may be required to demonstrate significance. We also relied on self-reporting of CVD events and medication use by participants. Hence, there could be recall and misclassification bias. Lastly, these biomarkers are dynamic in acute events, such as stroke and AMI [12,50,51]; however, the time from the event to the biomarker collection was not ascertained in this study. 

Despite healthcare advancements and the availability of state-of-the-art medical care, the burden of AMI and stroke continues to increase with increasing prevalence of chronic metabolic disease and an aging population, resulting in rising healthcare costs in many developed countries, including the United States [52], United Kingdom [53], and Singapore [54]. Given that the majority of CVDs are preventable, there is substantial opportunity to make a difference. Based on our findings, recommendations for CVD prevention strategies remain unchanged with emphasis on early identification and management of risk factors. 

This study adds to the current understanding of the factors that influence these biomarkers and found no correlation between them and CVD. However, more exploratory research is needed to continue the search for alternative biomarkers implicated in atherosclerosis for earlier detection and as potential therapeutic targets for CVD. Additionally, studies on these four biomarkers, RANTES, EMMPRIN, MMP-2, and MMP-9, should be conducted in better-defined demographic groups and in the setting of acute cardiovascular events to elucidate the impact and relationship between the biomarkers and CVD. A longitudinal follow-up of this cohort may help assess the predictive value of these biomarkers, such as recurrent myocardial infarctions, stroke, and mortality.

## Figures and Tables

**Table 1:** Characteristics of the study population.

	Total	Case (with CVD)	Control (without CVD)	*p*-Value *
	N = 515	N = 254	N = 261	
Females, n (%)	178 (34.6%)	89 (35.0%)	89 (34.1%)	0.820
Age	55.65 (10.31)	55.5827 (10.36)	55.7126 (10.27)	0.890
Ethnicity, n (%)				0.960
Chinese	331 (64.3%)	163 (64.2%)	168 (64.4%)	
Malay	73 (14.2%)	37 (14.6%)	36 (13.8%)	
Indian	111 (21.6%)	54 (21.3%)	57 (21.8%)	
logRANTES	3.75 (0.43)	3.76 (0.43)	3.74 (0.43)	0.730
logEMMPRIN	1.55 (0.24)	1.56 (0.23)	1.54 (0.25)	0.340
logMMP-2	5.39 (0.24)	5.38 (0.25)	5.39 (0.23)	0.710
logMMP-9	5.79 (0.59)	5.80 (0.58)	5.79 (0.60)	0.770
Current/previous smoker, n (%)	140 (27.2%)	78 (30.7%)	62 (23.8%)	0.076
Hypertension, n (%)	191 (37.1%)	119 (46.9%)	72 (27.6%)	<0.001
Hyperlipidemia, n (%)	197 (38.3%)	112 (44.1%)	85 (32.6%)	0.007
Diabetes Mellitus, n (%)	84 (16.3%)	55 (21.7%)	29 (11.1%)	0.001
Metabolic Syndrome, n (%)	158 (30.7%)	87(34.3%)	71(27.2%)	0.083
Framingham score	0.18 (0.15)	0.20 (0.16)	0.16 (0.14)	0.004
Gout, n (%)	38 (7.4%)	21 (8.3%)	17 (6.5%)	0.450
Chronic kidney disease, n (%)	6 (1.2%)	5 (2.0%)	1 (0.4%)	0.150
Sedentary time total hrs/week	38.58 (18.58)	39.64 (18.67)	37.56 (18.47)	0.200
Family history, n (%)	137 (26.6%)	76 (29.9%)	61 (23.4%)	0.093
Waist, cm	87.86 (11.48)	89.30 (11.34)	86.46 (11.46)	0.005
BMI, kg/m²	25.45 (4.26)	26.06 (4.41)	24.86 (4.03)	0.001
SBP, mmHg	129.66 (18.38)	132.13 (18.87)	127.26 (17.59)	0.003
DBP, mmHg	77.04 (11.56)	77.82 (12.22)	76.28 (10.86	0.130
LVH by ECG, n (%)	17(3.3%)	11(4.3%)	6 (2.3%)	0.200
TC, mmol/L	5.11 (0.94)	5.10 (0.97)	5.12 (0.91)	0.790
HDL, mmol/L	1.27 (0.31)	1.25 (0.27)	1.30 (0.34)	0.066
LDL, mmol/L	3.16 (0.82)	3.16 (0.86)	3.16 (0.77)	1
Cholesterol Ratio	4.18 (1.00)	4.24 (1.03)	4.12 (0.99)	0.18
Triglycerides, mmol/L	1.56 (0.85)	1.61 (0.91)	1.52 (0.80)	0.22
Creatinine, μmol/L	75.11 (22.28)	77.39 (26.80)	72.89 (16.52)	0.022
eGFR, mL/min/1.73m^2,^	91.96 (27.35)	92.93 (29.66)	91.01 (24.93)	0.430
Hs-CRP, mg/L	2.81 (6.64)	3.51 (8.97)	2.14 (2.81)	0.019
HbA1c %	6.10 (1.29)	6.31(1.46)	5.90 (1.07)	<0.001
Lipid lowering medications, n (%)	146(28.3)	91(35.8)	55(21.1)	<0.001
Hypertensive medications, n (%)	151(29.3)	96(37.8)	55(21.1)	<0.001

Data are presented as mean (SD) for continuous variables unless stated otherwise as n (%) for categorical variables. * *p*-value was obtained via chi-square for categorical variables and Student’s *t*-tests for continuous variables with a *p*-value of <0.05 taken to be statistically significant. BMI = body mass index, CKD = chronic kidney disease, cholesterol ratio = TC/LDL, DBP = diastolic blood pressure, EMMPRIN = extracellular matrix metalloproteinase inducer eGFR = estimated glomerular filtration rate, Framingham score = algorithm used to estimate the 10-year CVD risk of an individual. HbA1c = glycated hemoglobin, HDL = high-density lipoprotein, Hs-CRP = Highly sensitive C-reactive protein, LDL = low-density lipoprotein, LVH = left ventricular hypertrophy, MMP = matrix metalloproteinases, RANTES = Regulated on Activation, Normal T Cell Expressed and Secreted, SBP = systolic blood pressure, TC = total cholesterol.

**Table 2:** Factors associated with the biomarkers: (**a**): LogRANTES; (**b**): Log EMMPRIN; (**c**): Log MMP-2; (**d**): Log MMP-9.

Variable	(a) logRANTES														
	Model 1.0			Model 1.1			Model 1.2			Model 1.3			Model 1.4		
	β	95% CI	*p*	β	95% CI	*p*	β	95% CI	*p*	β	95% CI	*p*	β	95% CI	*p*
CVD composite	0.013	(−0.061, 0.087)	0.731												
CAD	0.078	(−0.089,0.082)	0.195										0.008	(−0.088, 0.103)	0.875
Arrhythmia	0.048	(−0.262, 0.165)	0.656										−0.008	(−0.223, 0.207)	0.943
Stroke	0.078	(−0.40, 0.196)	0.195										0.095	(−0.037, 0.226)	0.159
Age	−0.04	(−0.076, −0.004)	0.029	−0.042	(−0.079, −0.004)	0.029	−0.042	(−0.078, −0.006)	0.021	−0.043	(−0.080, −0.007)	0.021	−0.036	(−0.076, 0.004)	0.08
Male	ref												ref		
Female	0.153	(0.077, 0.230)	<0.001	0.141	(0.06, 0.221)	0.001	0.167	(0.090, 0.243)	<0.001	0.212	(0.109, 0.314)	<0.001	0.189	(0.078, 0.300)	0.001
Ethnicity			0.055 *						0.213 *			0.268 *			0.392*
Chinese	ref						ref			ref			ref		
Malay	0.059	(−0.049, 0.167)	0.282				0.013	(−0.095, 0.121)	0.814	0.002	(−0.109, 0.113)	0.976	−0.007	(−0.131, 0.117)	0.911
Indian	0.109	(0.017, 0.201)	0.02				0.083	(−0.010,0.176)	0.081	0.076	(−0.019, 0.172)	0.118	0.067	(−0.036, 0.169)	0.204
Gout	−0.105	(−0.246, 0.036)	0.143												
CKD	−0.113	(−0.458, 0.232)	0.519							−0.109	(−0.466, 0.248)	0.548	−0.168	(−0.617, 0.281)	0.463
Family History	−0.045	(−0.129, 0.038)	0.288												
Sedentary time,	0.001	(−0.001, 0.003)	0.279										0.001	(−0.001, 0.004)	0.209
Hrs/week															
BMI kg/m²	0.01	(0.001, 0.018)	0.03							0.004	(−0.006, 0.014)	0.444	0.003	(−0.009, 0.014)	0.652
Metabolic Syndrome	0.025	(−0.055, 0.105)	0.542							−0.022	(−0.111, 0.066)	0.621	−0.01	(−0.108, 0.088)	0.84
LVH ECG	−0.161	(−0.368, 0.046)	0.127												
Framingham score	−0.405	(−0.645, −0.165)	0.001												
LDL mmol/L	0.017	(−0.119, 0.153)	0.804										0.041	(−0.102, 0.184)	0.575
Creatinine mg/L	−0.133	(−0.285, 0.183)	0.085							0.136	(−0.071, 0.343)	0.198	0.131	(−0.094, 0.355)	0.254
Hs−CRP mg/L	0.044	(0.012, 0.077)	0.008	0.041	(0.007, 0.075)	0.019	0.035	(0.001, 0.068)	0.041	0.031	(−0.004, 0.067)	0.083	0.034	(−0.004, 0.071)	0.081
Hba1c %	0.096	(−0.112, 0.304)	0.366												
Lipid lowering medications	0.018	(−0.064, 0.100)	0.669												
Hypertensive medications	0.022	(−0.060, 0.103)	0.6												
**Variable**	**(b) logEMMPRIN**														
	**Model1.0**			**Model1.1**			**Model 1.2**			**Model 1,3**			**Model 1.4**		
	**β**	**95% CI**	***p***	**β**	**95% CI**	***p***	**β**	**95% CI**	***p***	**β**	**95% CI**	***p***	**β**	**95% CI**	***p***
CVD composite	0.02	(−0.021, 0.062)	0.342												
CAD	0.011	(−0.037, 0.059)	0.641										0.012	(−0.042, 0.066)	0.66
Arrhythmia	−0.115	(−0.236, 0.005)	0.061	−0.113	(−0.232, 0.005)	0.061	−0.11	(−0.230, 0.009)	0.07	−0.11	(−0.230,0.009)	0.07	−0.124	(−0.245. −0.003	0.045
Stroke	0.075	(0.009, 0.141)	0.027	0.064	(−0.009, 0.137)	0.084	0.065	(−0.008, 0.139)	0.081	0.065	(−0.008,0.139)	0.081	0.06	(−0.014, 0.135)	0.11
Age	0.012	(−0.008, 0.032)	0.244				0.013	(−.008, 0.035)	0.232	0.012	(−0.010,0.034)	0.289	0.012	(−0.010, 0.035)	0.284
Male	ref						ref			ref			ref		
Female	0.006	(−0.037, 0.050)	0.774				0.021	(−0.025, 0.067)	0.359	0.02	(−0.026, 0.066)	0.402	0.042	(−0.020, 0.105)	0.185
Ethnicity			0.018 *						0.127 *			0.130 *			0.123 *
Chinese	ref						ref			ref			ref		
Malay	−0.004	(−0.065, 0.056)	0.887				−0.018	(−0.086, 0.050)	0.601	−0.019	(−0.087, 0.049)	0.578	−0.024	(−0.094, 0.046)	0.499
Indian	0.072	(0.021, 0.123)	0.006				0.051	( −0.006, 0.107)	0.078	0.05	(−0.007, 0.106)	0.084	0.048	(−0.010, 0.106)	0.105
Gout	0.055	(−0.024, 0.135)	0.172												
CKD	0.163	(−0.030, 0.357)	0.098	0.255	(0.021, 0.489)	0.033	0.258	(−0.006, 0.107)	0.031	0.257	(0.022,0.491)	0.032	0.216	(−0.037.0.469)	0.094
Family History	0.028	(−0.019, 0.075)	0.241												
Sedentary time,	−0.001	(−0.002, 0.001)	0.301							0	(−0.002, 0.001)	0.556	0	(−0.002, 0.001)	0.537
Hrs/week															
BMI kg/m²	0.003	(−0.002, 0.008)	0.222										0.002	(−0.004. 0.009)	0.459
Metabolic Syndrome	−0.017	(−0.062, 0.028)	0.453										−0.024	(−0.079, 0.031)	0.392
LVH ECG	−0.02	(−0.137, 0.096)	0.731												
Framingham score	0.048	(−0.089, 0.184)	0.491												
LDL mmol/L	0.109	(0.033, 0.184)	0.005	0.105	(0.027, 0.183)	0.009	0.112	(0.033, 0.191)	0.005	0.112	(0.034, 0.191)	0.005	0.111	(0.030, 0.191)	0.007
Creatinine mg/L	0.066	(−0.019, 0.151)	0.129										0.062	(−0.065, 0.188)	0.339
Hs−CRP mg/L	0.019	(0.001, 0.038)	0.04	0.02	(0.000, 0.039)	0.045	0.018	(−0.002, 0.038)	0.086	0.018	(−0.002, 0.038)	0.084	0.017	(−0.004, 0.038)	0.121
Hba1c %	0.013	(−0.104, 0.130)	0.827												
Lipid lowering medications	−0.022	(−0.069, 0.024)	0.342												
Hypertensive medications	−0.012	(−0.058, 0.033)	0.597												
**Variable**	**(c) logMMP2**														
	**Model 1.0**			**Model 1.1**			**Model 1.2**			**Model 1.3**			**Model 1.4**		
	**β**	**95% CI**	***p***	**β**	**95% CI**	***p***	**β**	**95% CI**	***p***	**β**	**95% CI**	***p***	**β**	**95% CI**	***p***
CVD composite	−0.008	(−0.049, 0.034)	0.713												
CAD	0.025	(−0.023,0.072)	0.311							0.027	(−0.021, 0.075)	0.269	0.031	(−0.022, 0.084)	0.256
Arrhythmia	0.004	(−0.114, 0.122)	0.953										0	(−0.120, 0.120)	0.998
Stroke	−0.031	(−0.096, 0.035)	0.359	−0.062	(−0.134, 0.010)	0.089	−0.038	(−0.103, 0.028)	0.26	−0.041	(−0.107, 0.025)	0.221	−0.065	(−0.138, 0.009)	0.084
Age	0.033	(0.013, 0.053)	0.001	0.032	(0.011, 0.053)	0.003	0.034	(0.014, 0.054)	0.001	0.036	(0.015, 0.056)	0.001	0.033	(0.010, 0.055)	0.004
Male	ref						ref			ref			ref		
Female	0.005	(−0.038, 0.049)	0.809				−0.001	(−0.044, 0.042)	0.963	0.001	(−0.043, 0.044)	0.982	0.013	(−0.049, 0.075)	0.684
Ethnicity			0.736 *						0.815 *			0.700 *			0.545 *
Chinese	ref						ref			ref			ref		
Malay	−0.023	(−0.084, 0.037)	0.454				−0.011	(−0.072, 0.050)	0.72	−0.026	(−0.090, 0.037)	0.418	−0.039	(−0.108, 0.031)	0.274
Indian	0.002	(−0.050, 0.053)	0.946				0.011	(−0.040, 0.063)	0.664	0	(−0.053, 0.053)	0.999	−0.006	(−0.063, 0.052)	0.849
Gout	0.011	(−0.068, 0.090)	0.782												
CKD	−0.011	(−0.203, 0.181)	0.909										−0.036	(−0.286, 0.214)	0.777
Family History	−0.025	(−0.072, 0.022)	0.294												
Sedentary time,	0.001	(−0.001, 0.002)	0.327										0.001	(−0.001, 0.002)	0.371
Hrs/week															
BMI kg/m²	0.002	(−0.003, 0.007)	0.361							0.004	(−0.001, 0.009)	0.164	0.005	(−0.002, 0.011)	0.144
Metabolic Syndrome	0.022	(−0.023, 0.067)	0.336										0.01	(−0.044,0.65)	0.715
LVH ECG	0.013	(−0.103, 0.128)	0.829												
Framingham score	0.157	(0.023, 0.292)	0.022												
LDL mmol/L	−0.013	(−0.088, 0.063)	0.739							0.006	(−0.070, 0.082)	0.873	0.006	(−0.073, 0.086)	0.873
Creatinine mg/L	0.002	(−0.082, 0.087)	0.956										−0.005	(−0.130, 0.120)	0.938
Hs−CRP mg/L	−0.015	(−0.034, 0.003)	0.101										−0.019	(−0.040, 0.002)	0.072
Hba1c %	0.021	(−0.095, 0.137)	0.725												
Lipid lowering medications	0.012	(−0.033, 0.058)	0.598												
Hypertensive medications	0.024	(−0.021, 0.070)	0.29												
**Variable**	**(d) logMMP9**														
	**Model1.0**			**Model 1.1**			**Model 1.2**			**Model 1.3**			**Model 1.4**		
	**β**	**95% CI**	***p***	**β**	**95% CI**	***p***	**β**	**95% CI**	***p***	**β**	**95% CI**	***p***	**β**	**95% CI**	***p***
CVD composite	0.013	(−0.088, 0.118)	0.731												
CAD	0.033	(−0.085, 0.152)	0.583							−0.002	(−0.120, 0.115)	0.972	0.035	(−0.097, 0.168)	0.6
Arrhythmia	−0.279	(−0.579, 0.020)	0.068										−0.256	(−0.082, 0.286)	0.094
Stroke	0.108	(−0.055, 0.272)	0.194										0.102	(−0.556, 0.044)	0.275
Age	−0.015	(−0.065, 0.035)	0.549				0.005	(−0.044, 0.055)	0.829	0.008	(−0.043, 0.058)	0.767	0.02	(−0.036, 0.075)	0.49
Male	ref			ref			ref			ref			ref		
Female	−0.199	(−0.306, −0.092)	<0.001	−0.186	(−0.298, −0.074)	0.001	−0.196	(−0.303, −0.090)	<0.001	−0.196	(−0.303, −0.089)	<0.001	−0.122	(−0.277, 0.033)	0.122
Ethnicity			0.029 *			0.043 *			0.184 *			0.208 *			0.032*
Chinese	ref			ref		0.208	ref			ref			ref		
Malay	0.05	(−0.100, 0.199)	0.515	0.072	(−0.092, 0.236)	0.388	0.03	(−0.121, 0.181)	0.698	0.018	(−0.139, 0.174)	0.823	0.075	(−0.099, 0.248)	0.397
Indian	0.173	(0.046, 0.300)	0.008	0.175	(0.037, 0.313)	0.013	0.122	(−0.008, 0.251)	0.066	0.118	(−0.014, 0.250)	0.08	0.192	(0.048, 0.335)	0.009
Gout	0.004	(−0.192, 0.201)	0.966												
CKD	0.249	(−0.229, 0.728)	0.306										−0.044	(−0.670, 0.583)	0.892
Family History	−0.06	(−0.176, 0.056)	0.313												
Sedentary time,	0.002	(−0.001, 0.005)	0.115										0.002	(−0.001, 0.005)	0.258
Hrs/week															
BMI kg/m²	0.009	(−0.003, 0.021)	0.139							0.001	(−0.012, 0.014)	0.899	0.003	(−0.013, 0.019)	0.681
Metabolic Syndrome	−0.026	(−0.137, 0.086)	0.649										−0.104	(−0.241, 0.033)	0.136
LVH ECG	−0.062	(−0.350, 0.225)	0.67												
Framingham score	0.227	(−0.109, 0.562)	0.185												
LDL mmol/L	0.104	(−0.084, 0.292)	0.278							0.103	(−0.085, 0.290)	0.283	0.094	(−0.105, 0.294)	0.353
Creatine mg/L	0.316	(0.107, 0.525)	0.003										0.134	(−0.179, 0.447)	0.401
Hs−CRP mg/L	0.072	(0.027, 0.118)	0.002	0.06	(0.010, 0.110)	0.019	0.063	(0.017, 0.110)	0.008	0.063	(0.014, 0.112)	0.012	0.063	(0.011, 0.116)	0.019
Hba1c %	0.079	(−0.210, 0.367)	0.591												
Lipid lowering medications	−0.097	(−0.210, 0.017)	0.095												
Hypertensive medications	−0.031	(−0.144, 0.082)	0.589												

(a–d) *p*-Value of <0.05 was taken to be statistically significant. BMI = body mass index, CAD = coronary artery disease, CKD = chronic kidney disease, CAD = coronary artery disease, CVD = cardiovascular disease, Framingham score = algorithm used to estimate the 10-year CVD risk of an individual. HbA1c =glycated hemoglobin, Hs-CRP = highly sensitive C-reactive protein, LDL = low-density lipoprotein, LVH = left ventricular hypertrophy. *: *p*-value to assess whether at least one of the β coefficients associated with ethnicity is non-zero. Model 1 = forward stepwise selection. Model 2 = Model 1 + age, gender, and ethnicity. (a) Model 3 = Model 2 + previously published associations (CKD, BMI, metabolic syndrome, creatinine). (b) Model 3 = Model 2 + previously published associations (sedentary time). (c,d) Model 3 = Model 2 + previously published associations (CAD, BMI, LDL). Model 4 = all factors that appeared in Model 3 of the biomarkers but are shared by all four biomarkers (age, gender, ethnicity, BMI, CAD, stroke/TIA, arrhythmia, CKD, metabolic syndrome, sedentary time, LDL, creatinine).

**Table 3:** Association of inflammatory biomarkers with CVD.

	log RANTES	log EMMPRIN	log MMP-2	log MMP-9
	OR, 95% CI	OR, 95% CI	OR, 95% CI	OR, 95% CI
**Univariate**	1.074	1.421	0.872	1.044
	(0.716, 1.700)	(0.688, 2.936)	(0.422, 1.803)	(0.780–1.397)
**Model 2.1**	1.064	1.441	0.876	1.009
	(0.703, 1.609)	(0.480, 1.720)	(0.421, 1.826)	(0.749, 1.358)
**Model 2.2**	1.067	1.471	0.879	1.013
	(0.704, 1.617)	(0.702, 3.084)	(0.422, 1.832)	(0.750, 1.369)
**Model 2.3**	0.937	1.348	0.834	1.046
	(0.602, 1.457)	(0.610, 2.980)	(0.384, 1.810)	(0.762, 1.436)
**Model 2.4**	0.934	1.348	0.836	1.044
	(0.601, 1.452)	(0.610, 2.980)	(0.385, 1.817)	(0.760, 1.433)

Model 2.1 = adjusted for significant biomarker factors from Table 2. Model 2.2 = Model 1+ adjusted for age, sex, and ethnicity. Model 2.3 = Model 2 + other significant variables from Table 1(hypertension, hyperlipidemia, diabetes, Framingham score, BMI, waist, mean SBP, creatinine, hba1c, hs-CRP) Model 2.4 = all factors that appeared in Model 3 of the biomarkers but are shared by all four biomarkers (age, sex, ethnicity, hypertension, hyperlipidemia, diabetes, Framingham score, BMI, waist, mean SBP, creatinine, hba1c, LDL, hs-CRP).

## Data Availability

Data described in the manuscript, code book, and analytic code will be made available upon request pending application and approval from the corresponding author.

## Supplementary Materials

The following supporting information can be downloaded at: https://www.mdpi.com/article/10.3390/jcm11247281/s1, Table S1: Sensitivity analysis for log MMP-2 excluding pre-menopausal women; Table S2: Sensitivity analysis for log MMP-9 excluding pre-menopausal women.

## Abbreviations and Acronyms

EMMPRIN:	extracellular matrix metalloproteinase inducer
FRS:	Framingham risk score
Hs-CRP:	highly sensitive C-reactive protein
MEC:	multi-ethnic cohort (MEC)
MMP:	matrix metalloproteinases
NLR:	neutrophil-to-lymphocyte ratio
PLR:	platelet-to-lymphocyte ratio
RANTES:	regulated on Activation, Normal T Cell Expressed and Secreted
SP2:	Singapore Prospective Study Program
SCCS2:	Singapore Cardiovascular Cohort Study
SII:	systemic inflammation index

## References

[1] WHO (2017). Cardiovascular Diseases (CVDs). https://www.who.int/en/news-room/fact-sheets/detail/cardiovascular-diseases-(cvds).

[2] Tsao C.W., Aday A.W., Almarzooq Z.I., Alonso A., Beaton A.Z., Bittencourt M.S., Boehme A.K., Buxton A.E., Carson A.P., Commodore-Mensah Y. (2022). Heart Disease and Stroke Statistics-2022 Update: A Report From the American Heart Association. Circulation.

[3] Abete P., della Morte D., Gargiulo G., Basile C., Langellotto A., Galizia G., Testa G., Canonico V., Bonaduce D., Cacciatore F. (2014). Cognitive impairment and cardiovascular diseases in the elderly. A heart-brain continuum hypothesis. Ageing Res. Rev..

[4] Weber C., Noels H. (2011). Atherosclerosis: Current pathogenesis and therapeutic options. Nat. Med..

[5] Tsimikas S., Willerson J.T., Ridker P.M. (2006). C-reactive protein and other emerging blood biomarkers to optimize risk stratification of vulnerable patients. J. Am. Coll. Cardiol..

[6] Soeki T., Sata M. (2016). Inflammatory Biomarkers and Atherosclerosis. Int. Heart J..

[7] Lipkova J., Parenica J., Duris K., Helanova K., Tomandl J., Kubkova L., Vasku A., Goldbergova Pavkova M. (2015). Association of circulating levels of RANTES and -403G/A promoter polymorphism to acute heart failure after STEMI and to cardiogenic shock. Clin. Exp. Med..

[8] Appay V., Rowland-Jones S.L. (2001). RANTES: A versatile and controversial chemokine. Trends Immunol..

[9] Cavusoglu E., Eng C., Chopra V., Clark L.T., Pinsky D.J., Marmur J.D. (2007). Low plasma RANTES levels are an independent predictor of cardiac mortality in patients referred for coronary angiography. Arterioscler. Thromb. Vasc. Biol..

[10] Kaushik D.K., Hahn J.N., Yong V.W. (2015). EMMPRIN, an upstream regulator of MMPs, in CNS biology. Matrix Biol..

[11] Agrawal S.M., Yong V.W. (2011). The many faces of EMMPRIN—Roles in neuroinflammation. Biochim. Et Biophys. Acta.

[12] Schmidt R., Bültmann A., Ungerer M., Joghetaei N., Bülbül O., Thieme S., Chavakis T., Toole B.P., Gawaz M., Schömig A. (2006). Extracellular matrix metalloproteinase inducer regulates matrix metalloproteinase activity in cardiovascular cells: Implications in acute myocardial infarction. Circulation.

[13] Opstad T.B., Seljeflot I., Bohmer E., Arnesen H., Halvorsen S. (2018). MMP-9 and Its Regulators TIMP-1 and EMMPRIN in Patients with Acute ST-Elevation Myocardial Infarction: A NORDISTEMI Substudy. Cardiology.

[14] Galis Z.S., Khatri J.J. (2002). Matrix metalloproteinases in vascular remodeling and atherogenesis: The good, the bad, and the ugly. Circ. Res..

[15] Newby A.C. (2015). Metalloproteinases promote plaque rupture and myocardial infarction: A persuasive concept waiting for clinical translation. Matrix Biol..

[16] Wang J., Tan G.J., Han L.N., Bai Y.Y., He M., Liu H.B. (2017). Novel biomarkers for cardiovascular risk prediction. J. Geriatr. Cardiol..

[17] Blankenberg S., Rupprecht H.J., Poirier O., Bickel C., Smieja M., Hafner G., Meyer J., Cambien F., Tiret L., AtheroGene Investigators (2003). Plasma concentrations and genetic variation of matrix metalloproteinase 9 and prognosis of patients with cardiovascular disease. Circulation.

[18] Kelly D., Cockerill G., Ng L.L., Thompson M., Khan S., Samani N.J., Squire I.B. (2007). Plasma matrix metalloproteinase-9 and left ventricular remodelling after acute myocardial infarction in man: A prospective cohort study. Eur. Heart J..

[19] Lenti M., Falcinelli E., Pompili M., de Rango P., Conti V., Guglielmini G., Momi S., Corazzi T., Giordano G., Gresele P. (2014). Matrix metalloproteinase-2 of human carotid atherosclerotic plaques promotes platelet activation. Correlation with ischaemic events. Thromb. Haemost..

[20] Dhillon O.S., Khan S.Q., Narayan H.K., Ng K.H., Mohammed N., Quinn P.A., Squire I.B., Davies J.E., Ng L.L. (2009). Matrix metalloproteinase-2 predicts mortality in patients with acute coronary syndrome. Clin. Sci..

[21] Tan K.H.X., Tan L.W.L., Sim X., Tai E.S., Lee J.J., Chia K.S., van Dam R.M. (2018). Cohort Profile: The Singapore Multi-Ethnic Cohort (MEC) study. Int. J. Epidemiol..

[22] D’Agostino R.B., Vasan R.S., Pencina M.J., Wolf P.A., Cobain M., Massaro J.M., Kannel W.B. (2008). General cardiovascular risk profile for use in primary care: The Framingham Heart Study. Circulation.

[23] (2002). Third Report of the National Cholesterol Education Program (NCEP) Expert Panel on Detection, Evaluation, and Treatment of High Blood Cholesterol in Adults (Adult Treatment Panel III) final report. Circulation.

[24] Yao L., Herlea-Pana O., Heuser-Baker J., Chen Y., Barlic-Dicen J. (2014). Roles of the Chemokine System in Development of Obesity, Insulin Resistance, and Cardiovascular Disease. J. Immunol. Res..

[25] Rothenbacher D., Müller-Scholze S., Herder C., Koenig W., Kolb H. (2006). Differential Expression of Chemokines, Risk of Stable Coronary Heart Disease, and Correlation with Established Cardiovascular Risk Markers. Arterioscler. Thromb. Vasc. Biol..

[26] Mikolajczyk T.P., Szczepaniak P., Vidler F., Maffia P., Graham G.J., Guzik T.J. (2021). Role of inflammatory chemokines in hypertension. Pharmacol. Ther..

[27] Wang C.-H., Dai J.-Y., Wang L., Jia J.-F., Zheng Z.-H., Ding J., Chen Z.-N., Zhu P. (2011). Expression of CD147 (EMMPRIN) on neutrophils in rheumatoid arthritis enhances chemotaxis, matrix metalloproteinase production and invasiveness of synoviocytes. J. Cell. Mol. Med..

[28] von Ungern-Sternberg S.N.I., Zernecke A., Seizer P. (2018). Extracellular Matrix Metalloproteinase Inducer EMMPRIN (CD147) in Cardiovascular Disease. Int. J. Mol. Sci..

[29] Elahirad S., Elieh Ali Komi D., Kiani A., Mohammadi-Noori E., Vaisi-Raygani A., Mozafari H., Bahrehmand F., Saidi M., Toupchi-Khosroshahi V., Salehi N. (2022). Association of Matrix Metalloproteinase-2 (MMP-2) and MMP-9 Promoter Polymorphisms, Their Serum Levels, and Activities with Coronary Artery Calcification (CAC) in an Iranian Population. Cardiovasc. Toxicol..

[30] Derosa G., Maffioli P., D’Angelo A., Salvadeo S.A., Ferrari I., Fogari E., Gravina A., Mereu R., Palumbo I., Randazzo S. (2009). Evaluation of metalloproteinase 2 and 9 levels and their inhibitors in combined dyslipidemia. Clin. Investig. Med..

[31] Boumiza S., Chahed K., Tabka Z., Jacob M.-P., Norel X., Ozen G. (2021). MMPs and TIMPs levels are correlated with anthropometric parameters, blood pressure, and endothelial function in obesity. Sci. Rep..

[32] Salamonsen L.A., Woolley D.E. (1996). Matrix metalloproteinases in normal menstruation. Hum. Reprod..

[33] Ueba T., Nomura S., Inami N., Yokoi T., Inoue T. (2014). Elevated RANTES level is associated with metabolic syndrome and correlated with activated platelets associated markers in healthy younger men. Clin. Appl. Thromb. Hemost..

[34] Herder C., Peeters W., Illig T., Baumert J., de Kleijn D.P., Moll F.L., Poschen U., Klopp N., Müller-Nurasyid M., Roden M. (2011). RANTES/CCL5 and risk for coronary events: Results from the MONICA/KORA Augsburg case-cohort, Athero-Express and CARDIoGRAM studies. PLoS ONE.

[35] Liu H., Yang L.X., Guo R.W., Zhu G.F., Shi Y.K., Wang X.M., Qi F., Guo C.M., Ye J.S., Yang Z.H. (2013). Functional blockage of EMMPRIN ameliorates atherosclerosis in apolipoprotein E-deficient mice. Int. J. Cardiol..

[36] Kjekshus H., Skrebelyte-Strom L., Bakkelund V., Arnesen H., Ronning O.M., Steine K., Seljeflot I. (2020). Biomarkers in patients with cryptogenic stroke/TIA and subclinical atrial fibrillation. Eur. Heart J..

[37] Yasmin, Wallace S., McEniery C.M., Dakham Z., Pusalkar P., Maki-Petaja K., Ashby M.J., Cockcroft J.R., Wilkinson I.B. (2005). Matrix Metalloproteinase-9 (MMP-9), MMP-2, and Serum Elastase Activity Are Associated With Systolic Hypertension and Arterial Stiffness. Arterioscler. Thromb. Vasc. Biol..

[38] Porhcisaliyan V.D., Wang Y., Tan N.C., Jafar T.H. (2021). Socioeconomic status and ethnic variation associated with type 2 diabetes mellitus in patients with uncontrolled hypertension in Singapore. BMJ Open Diabetes Res. Care.

[39] Lee J., Heng D., Chia K.S., Chew S.K., Tan B.Y., Hughes K. (2001). Risk factors and incident coronary heart disease in Chinese, Malay and Asian Indian males: The Singapore Cardiovascular Cohort Study. Int. J. Epidemiol..

[40] Gibson A.O., Blaha M.J., Arnan M.K., Sacco R.L., Szklo M., Herrington D.M., Yeboah J. (2014). Coronary artery calcium and incident cerebrovascular events in an asymptomatic cohort. The MESA Study. JACC Cardiovasc. Imaging.

[41] Ridker P.M., Everett B.M., Pradhan A., MacFadyen J.G., Solomon D.H., Zaharris E., Mam V., Hasan A., Rosenberg Y., Iturriaga E. (2019). Low-Dose Methotrexate for the Prevention of Atherosclerotic Events. N. Engl. J. Med..

[42] Ridker P.M., Everett B.M., Thuren T., MacFadyen J.G., Chang W.H., Ballantyne C., Fonseca F., Nicolau J., Koenig W., Anker S.D. (2017). Antiinflammatory Therapy with Canakinumab for Atherosclerotic Disease. N. Engl. J. Med..

[43] Pasqui E., de Donato G., Brancaccio B., Casilli G., Ferrante G., Cappelli A., Palasciano G. (2022). The Predictive Role of Inflammatory Biochemical Markers in Post-Operative Delirium After Vascular Surgery Procedures. Vasc. Health Risk Manag..

[44] Tamhane U.U., Aneja S., Montgomery D., Rogers E.K., Eagle K.A., Gurm H.S. (2008). Association between admission neutrophil to lymphocyte ratio and outcomes in patients with acute coronary syndrome. Am. J. Cardiol..

[45] Taurino M., Aloisi F., Del Porto F., Nespola M., Dezi T., Pranteda C., Rizzo L., Sirignano P. (2021). Neutrophil-to-Lymphocyte Ratio Could Predict Outcome in Patients Presenting with Acute Limb Ischemia. J. Clin. Med..

[46] Pasqui E., de Donato G., Lazzeri E., Molino C., Galzerano G., Giubbolini M., Palasciano G. (2022). High Neutrophil-to-Lymphocyte and Platelet-to-Lymphocyte Ratios Are Associated with a Higher Risk of Hemodialysis Vascular Access Failure. Biomedicines.

[47] Iyer R.P., Patterson N.L., Fields G.B., Lindsey M.L. (2012). The history of matrix metalloproteinases: Milestones, myths, and misperceptions. Am. J. Physiol. Circ. Physiol..

[48] Halade G.V., Jin Y.F., Lindsey M.L. (2013). Matrix metalloproteinase (MMP)-9: A proximal biomarker for cardiac remodeling and a distal biomarker for inflammation. Pharmacol. Ther..

[49] Virani S.S., Nambi V., Hoogeveen R., Wasserman B.A., Coresh J., Gonzalez F., Chambless L.E., Mosley T.H., Boerwinkle E., Ballantyne C.M. (2011). Relationship between circulating levels of RANTES (regulated on activation, normal T-cell expressed, and secreted) and carotid plaque characteristics: The Atherosclerosis Risk in Communities (ARIC) Carotid MRI Study. Eur. Heart J..

[50] Tokami H., Ago T., Sugimori H., Kuroda J., Awano H., Suzuki K., Kiyohara Y., Kamouchi M., Kitazono T. (2013). RANTES has a potential to play a neuroprotective role in an autocrine/paracrine manner after ischemic stroke. Brain Res..

[51] Chihara J., Yasuba H., Tsuda A., Urayama O., Saito N., Honda K., Kayaba H., Yamashita T., Kurimoto F., Yamada H. (1997). Elevation of the plasma level of RANTES during asthma attacks. J. Allergy Clin. Immunol..

[52] Birger M., Kaldjian A.S., Roth G.A., Moran A.E., Dieleman J.L., Bellows B.K. (2021). Spending on Cardiovascular Disease and Cardiovascular Risk Factors in the United States: 1996 to 2016. Circulation.

[53] Public Health England. https://www.gov.uk/government/publications/health-matters-preventing-cardiovascular-disease/health-matters-preventing-cardiovascular-disease.

[54] (2020). Principal Causes of Death: Ministry of Health. https://www.moh.gov.sg/resources-statistics/singapore-health-facts/principal-causes-of-death.

