# Supplementary material for: Factors Associated with RANTES, EMMPIRIN, MMP2 and MMP9, and the Association of These Biomarkers with Cardiovascular Disease in a Multi-Ethnic Population
Source: J Clin Med. 2022 Dec 8;11(24):7281. doi: 10.3390/jcm11247281 (PMC9782242; doi:10.3390/jcm11247281)
Supplement: Supplementary file 1 [file jcm-11-07281-s001.zip › jcm-2015589-supplementary.pdf]

Journal of
Clinical Medicine

MDPI

Supplemental material

Table S1: Sensitivity analysis for log MMP-2 excluding pre-menopausal women

Variable	logMMP2														
	Model 1.0			Model 1.1			Model 1.2			Model 1.3			Model 1.4		
	β	95%CI	p	β	95%CI	p	β	95%CI	p	β	95%CI	p	β	95%CI	p
**CVD composite**	-0.005	(-0.047, 0.036)	0.797												
**CAD**	0.024	(-0.024, 0.072)	0.327							0.278	(-0.021, 0.076)	0.260	0.033	(-0.021, 0.087)	0.225
**Arrhythmia**	0.011	(-0.116, 0.137)	0.869										-0.006	(-0.134, 0.122)	0.924
**Stroke**	-0.032	(-0.099, 0.036)	0.356	-0.040	(-0.107, 0.027)	0.243	-0.040	(-0.108, 0.027)	0.244	-0.044	(-0.112, 0.024)	0.204	-0.070	(-0.146, 0.006)	0.071
**Age**	0.031	(0.010, 0.052)	0.005	0.032	(0.011, 0.053)	0.003	0.031	(0.009, 0.053)	0.006	0.033	(0.011, 0.055)	0.004	0.031	(0.007, 0.055)	0.011
**Male**	ref						ref			ref			ref		
**Female**	0.018	(-0.027, 0.063)	0.435				0.005	(-0.041, 0.051)	0.830	0.006	(-0.040, 0.052)	0.795	0.020	(-0.044, 0.084)	0.536
**Ethnicity**			0.865*						0.923*			0.700*			*0.545
**Chinese**	ref						ref			ref			ref		
**Malay**	-0.017	(-0.079, 0.045)	0.592				-0.008	(-0.071, 0.054)	0.791	-0.026	(-0.092, 0.039)	0.427	-0.046	(-0.118, 0.025)	0.206
**Indian**	-0.002	(-0.054, 0.051)	0.954				0.006	(-0.046, 0.057)	0.815	-0.007	(-0.061, 0.048)	0.809	-0.013	(-0.072, 0.045)	0.653
**Gout**	0.007	(-0.072, 0.086)	0.863												
**CKD**	-0.015	(-0.207, 0.177)	0.877										-0.046	(-0.297, 0.205)	0.717
**Family History**	-0.027	(-0.075, 0.020)	0.256												
**Sedentary time,** Hrs/week	0.001	(-0.001, 0.002)	0.232										0.001	(-0.001, 0.002)	0.339
**BMI** kg/m²	0.003	(-0.002, 0.008)	0.238							0.004	(-0.001, 0.009)	0.112	0.005	(-0.002, 0.011)	0.140
**Metabolic Syndrome**	0.021	(-0.025, 0.066)	0.373										0.007	(-0.049, 0.062)	0.813
**LVH ECG**	0.009	(-0.107, 0.124)	0.883												
**Framingham score**	0.136	(-0.001, 0.273)	0.051												
**LDL** mmol/L	-0.014	(-0.090, 0.063)	0.722							0.004	(-0.074, 0.081)	0.926	0.002	(-0.079, 0.083)	0.962
**Creatinine** mg/L	-0.006	(-0.092, 0.080)	0.895										0.007	(-0.119, 0.134)	0.911
**Hs-CRP** mg/L	-0.013	(-0.031, 0.006)	0.190										-0.016	(-0.038, 0.005)	0.143
**Hba1c** %	0.009	(-0.108, 0.125)	0.885												
**Lipid lowering medications**	0.007	(-0.039, 0.053)	0.767												
**Hypertensive medications**	0.019	(-0.026, 0.065)	0.409												

P-value of <0.05 was taken to be statistically significant.

BMI=body mass index, CAD= coronary artery disease, CKD=chronic kidney disease, CAD=coronary artery disease, CVD=cardiovascular disease, Framingham score = algorithm used to estimate the 10-year CVD risk of an individual. HbA1c =glycated hemoglobin, Hs-CRP=Highly sensitive C reactive protein, LDL=low density lipoprotein, LVH=Left ventricular hypertrophy.

*: p-value to assess whether at least one of the β coefficients associated with ethnicity is non-zero.

The same models 1.1 to 1.4 from the main text was used for sensitivity analysis.

Table S2: Sensitivity analysis for log MMP-9 excluding pre-menopausal women

Variable	logMMP9														
	Model 1.0			Model 1.1			Model 1.2			Model 1.3			Model 1,4		
	β	95%CI	p	β	95%CI	p	β	95%CI	p	β	95%CI	p	β	95%CI	p
**CVD composite**	0.023	(-0.081, 0.127)	0.660												
**CAD**	0.038	(-0.082, 0.157)	0.534							0.007	(-0.111, 0.125)	0.908	0.055	(-0.079, 0.189)	0.420
**Arrhythmia**	-0.261	(-0.580, 0.058)	0.109										-0.226	(-0.544, 0.091)	0.162
**Stroke**	0.099	(-0.068, 0.266)	0.245										0.086	(-0.103, 0.274)	0.371
**Age**	-0.002	(-0.055, 0.051)	0.938				0.033	(-0.021, 0.086)	0.230	0.035	(-0.019, 0.089)	0.201	0.045	(-0.015, 0.105)	0.140
**Male**	ref			ref			ref			ref			ref		
**Female**	-0.241	(-0.350, -0.132)	<0.001	-0.233	(-0.342, -0.125)	<0.001	-0.248	(-0.359, -0.137)	<0.001	-0.248	(-0.359, -0.136)	<0.001	-0.171	(-0.330, -0.011)	0.036
**Ethnicity**			0.027*			0.172*			0.148*			0.152*			0.030*
**Chinese**	ref			ref			ref			Ref			ref		
**Malay**	0.020	(-0.134, 0.174)	0.798	-0.008	(-0.161, 0.145)	0.919	0.004	(-0.150, 0.159)	0.956	-0.007	(-0.167, 0.153)	0.931	0.037	(-0.141, 0.215)	0.683
**Indian**	0.176	(0.047, 0.304)	0.008	0.119	(-0.011, 0.249)	0.072	0.128	(-0.003, 0.258)	0.055	0.125	(-0.009, 0.258)	0.067	0.194	(0.049, 0.339)	0.009
**Gout**	0.014	(-0.182, 0.210)	0.887												
**CKD**	0.258	(-0.218, 0.736)	0.287										-0.049	(-0.673, 0.574)	0.877
**Family History**	-0.065	(-0.183, 0.053)	0.280												
**Sedentary time,** Hrs/week	0.002	(-0.001, 0.005)	0.152										0.002	(-0.001, 0.005)	0.276
**BMI** kg/m²	0.008	(-0.003, 0.021)	0.139							0.0003	(-0.013, 0.014)	0.954	0.004	(-0.012, 0.021)	0.593
**Metabolic Syndrome**	-0.049	(-0.161, 0.064)	0.397										-0.122	(-0.261, 0.160)	0.083
**LVH ECG**	-0.053	(-0.340, 0.234)	0.717												
**Framingham score**	0.292	(-0.048, 0.632)	0.092												
**LDL** mmol/L	0.100	(-0.091, 0.290)	0.304							0.103	(-0.085, 0.291)	0.282	0.085	(-0.116, 0.286)	0.406
**Creatine** mg/L	0.363	(0.152, 0.574)	0.001										0.124	(-0.190, 0.438)	0.439
**Hs-CRP** mg/L	0.071	(0.025, 0.117)	0.003	0.063	(0.016, 0.110)	0.009	0.063	(0.016, 0.110)	0.008	0.063	(0.013 0.113)	0.013	0.064	(0.011, 0.118)	0.019
**Hba1c** %	0.101	(-0.189, 0.390)	0.496												
**Lipid lowering medications**	-0.085	(-0.200, 0.029)	0.144												
**Hypertensive medications**	-0.018	(-0.131, 0.095)	0.751												

P-value of <0.05 was taken to be statistically significant.

BMI=body mass index, CAD= coronary artery disease, CKD=chronic kidney disease, CAD=coronary artery disease, CVD=cardiovascular disease, Framingham score = algorithm used to estimate the 10-year CVD risk of an individual. HbA1c =glycated hemoglobin, Hs-CRP=Highly sensitive C reactive protein, LDL=low density lipoprotein, LVH=Left ventricular hypertrophy.

*: p-value to assess whether at least one of the β coefficients associated with ethnicity is non-zero.

The same models 1.1 to 1.4 from the main text was used for sensitivity analysis.
